# Autism spectrum heterogeneity: fact or artifact?

**DOI:** 10.1038/s41380-020-0748-y

**Published:** 2020-04-30

**Authors:** Laurent Mottron, Danilo Bzdok

**Affiliations:** 1 Department of Psychiatry, University of Montreal and Researcher for the CIUSSS-NIM, Rivière-des-Prairies Hospital, 7070, Perras Boul, Montreal, QC H1E 1A4 Canada; 2 Department of Biomedical Engineering, McConnell Brain Imaging Centre, Montreal Neurological Institute, Faculty of Medicine, McGill University, Montreal, QC H3A 2B4 Canada; 3 Mila - Quebec Artificial Intelligence Institute, Montreal, QC H2S 3H1 Canada

**Keywords:** Autism spectrum disorders, Neuroscience

## Abstract

The current diagnostic practices are linked to a 20-fold increase in the reported prevalence of ASD over the last 30 years. Fragmenting the autism phenotype into dimensional “autistic traits” results in the alleged recognition of autism-like symptoms in any psychiatric or neurodevelopemental condition and in individuals decreasingly distant from the typical population, and prematurely dismisses the relevance of a diagnostic threshold. Non-specific socio-communicative and repetitive DSM 5 criteria, combined with four quantitative specifiers as well as all their possible combinations, render limitless variety of presentations consistent with the categorical diagnosis of ASD. We propose several remedies to this problem: maintain a line of research on prototypical autism; limit the heterogeneity compatible with a categorical diagnosis to situations with a phenotypic overlap and a validated etiological link with prototypical autism; reintroduce the qualitative properties of autism presentations and of current dimensional specifiers, language, intelligence, comorbidity, and severity in the criteria used to diagnose autism in replacement of quantitative “social” and “repetitive” criteria; use these qualitative features combined with the clinical intuition of experts and machine-learning algorithms to differentiate coherent subgroups in today’s autism spectrum; study these subgroups separately, and then compare them; and question the autistic nature of “autistic traits”

## Introduction

The heterogeneity of autism is now universally accepted, at the phenotypic level under the DSM-5 term “spectrum”, as well as at the imaged brain [1] and etiology [2] levels. The overarching pervasive developmental disorders (PDD) category of the DSM-IV initiated a deviation towards less prototypical presentations of autism. Asperger’s syndrome was considered to be autism without the requirement of language signs and PDD not otherwise specified as subthreshold autism of various types. The current DSM-5 definition of autism spectrum disorder (ASD) merged the PDD subgroups *inter alia*, due to their poor inter-judge reliability and instability over time [3]. Doing away with PDD not otherwise specified as a category, which was responsible for the considerable increase of reported prevalence at the time, but for which the criteria were insufficiently reliable, [4] was expected to increase the specificity of the categorical diagnosis [5].

**Fig. 1:**
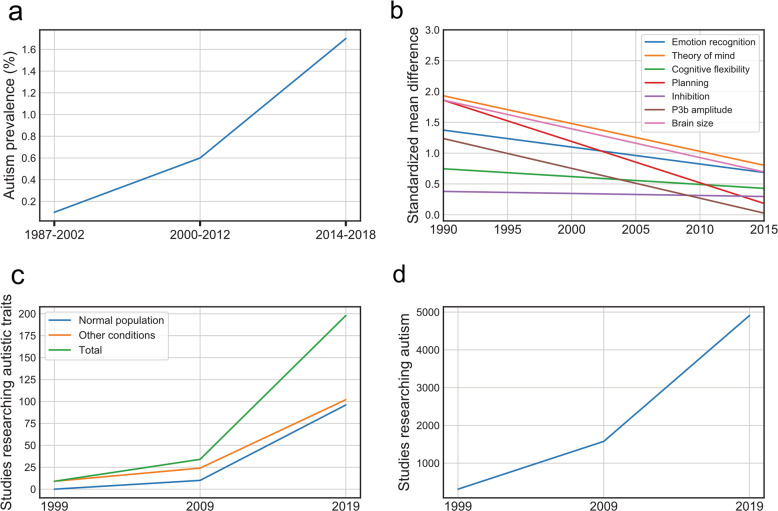
Temporal trends in autism research. **a** The change in autism prevalence over time, based on data from [65–67]. Methodologies may differ between studies. **b** The changes in group-level standardized mean differences between autism and control samples over time, as described by Rødgaard et al. (2019) [14]. A significant downwards temporal trend was observed in five of seven investigated constructs in autism. **c** The number of published studies investigating autistic traits in the normal population and for other clinical conditions, showing an eightfold increase during the last decade. **d** The number of published empirical studies performing research on autism, showing a fourfold increase during the last decade. (“**c**”,“**d**” source: Pubmed).

The evolution of the DSM has been accompanied by a 20-fold increase in the reported prevalence of ASD over the last 30 years, reaching a current prevalence of more than 2% in the United States [6] (Fig. 1a). The implementation of standardized retrospective [7] and observational diagnostic tools [8] in the diagnostic process has not limited this trend and may have even contributed to it in the clinical setting, possibly due to their lack of specificity towards other childhood psychiatric conditions [9] and the false sense of security they provide when someone “meets diagnostic criteria [10]”, despite clinical inconsistency with prototypical presentations.

A single categorical diagnosis, which encompasses such heterogeneity of developmental history, intelligence, comorbidity, and severity, poorly contributes to the planning of intervention and educational services. What is common between intervention strategies supporting an adult with academic-level written and oral language and an intellectually disabled, syndromic autistic child with major self-injurious behaviors [11]?

In addition to the clinical consequences, these considerations also have important ramifications for conducting and interpreting research studies. Currently, the modest state of genetic [12] knowledge of non-syndromic autism and the polygenic heterogeneity across ASD subtypes [13] suggests that we may need to update our research targets and strategies. The effect sizes obtained from cognitive, EEG, and neuro-anatomical studies of autism decreased by up to 80% between 2000 and 2015 [14] (Fig. 1b), even when accounting for sample size and quality. The cause of this trend is yet to be determined but likely includes a reduction in the deviation from the norm required to reach the threshold for a categorical diagnosis [15], and the poor specificity of current diagnostic criteria for extreme values of age, intelligence, and severity [16]. Other important factors that may contribute to this trend include the disappearance of differential exclusion diagnoses, as well as the absence of an “economy principle”, privileging the diagnosis which best explains the presented symptoms [17]. The presence of such “self-inflicted” heterogeneity plausibly distorts the autistic signal and negatively influences the ability to make replicable discoveries.

The wide-ranging disappearance of studies on differential diagnosis contrasts with the explosion of meta-analyses and systematic reviews (respectively less than 1/1000 vs. 3% of the autism literature in 2019). Most scientific knowledge of autism is obtained through condensing findings on an increasingly heterogeneous and decreasingly atypical population, without questioning the case ascertainment on which this knowledge is grounded. All research is carried out downstream of diagnostic criteria, little of it upstream. Sociologically, we are living in a time of rebalancing the traditional emphasis on stringent inclusion and exclusion criteria for clinical neuroscience studies. There is also growing interest in deriving insight from population and prospective cohorts with thousands of subjects [18]. As a necessary side effect of these developments in research trends, many autism-vs.-non-autism classification studies have become difficult to reproduce in these heterogeneous cohorts of unprecedented breadth [19]. We thus highlight the dilemma between the high predictive accuracies of preselected samples and much lower predictive accuracies of more naturally acquired subject samples [20]. In this context, the co-existence of qualitative and quantitative autism traits may have critical consequences for the future of single-patient predictions in precision psychiatry.

## Confronting essentialist and nominalist views of autism

We are still uncertain about the entity of autism. It can either appear to have a natural basis (“essentialist” position) or as multi-determinant, for which unity is in the eye of the viewer or the vocabulary they use (“nominalist” position). Although we are able to recognize this clinical condition with good reliability, we are not yet very skilled at defining it in a specific way. We also know that the signs that characterize it are not always simultaneously present, and/or that they can appear to be attenuated, making delineation of the autism category difficult.

However, the two positions should still compete for the truth. The absence of a categorical limit and the multiplicity of aetiologies and risk factors reported previously [21–23] anticipate the current state of knowledge. To be able to determine whether autism has a natural basis or not, the two positions should be given equal weight, as the validity of either is still undecidable in the current state of autism diagnosis. This requires studying individuals who are very similar to each other to obtain biomarkers, then to search for these markers in attenuated phenotypes. This does not guarantee that we will find a single basis in primary biology. However, if we start from the spectrum as currently defined, it is clear that we will not find this single cause, if it exists.

## DSM-5 criteria for autism may produce spurious heterogeneity

Combining nonspecific social and repetitive categorical criteria with four “open” specifiers (levels of intelligence, language, severity, and comorbidity), as well as all their possible combinations, can result in a vast array of ASD presentations. However, does such variability truthfully reflect diversity in cognition, the brain, and genes?

### The quantitative nature and poor specificity of signs which, when combined, result in a categorical diagnosis

The categorical diagnosis of ASD is currently obtained by pass/fail scoring of seven signs (i.e., three social and four repetitive), mostly quantitative (e.g., *less* socially-oriented behaviors), rather than qualitative (those that can be recognized). These signs are inherently imprecise due to their quantitative/dimensional nature (how do we define a threshold for “lack of socialization?”) and open character (from… to….), leading to the bundling of a variety of phenotypes that are not specific to autism. For example, autistic gaze atypicality and an embarrassed look are qualitatively distinct, but both make it possible to positively score the A2 criterion, “Deficits in nonverbal communicative behaviors used for social interaction”. Conversely, a rapid initial gaze at faces, followed by the apparent absence of behavioral hallmarks for social reciprocity, would become more specific by the addition of qualitative dimensions [24]. Certain signs (B2, “rigidity”, and B4, “sensory”), which when associated allow one to reach the diagnostic threshold in the area of repetitive behaviors, are also observed in a large proportion of children with other neurodevelopmental and psychiatric disorders [25, 26].

### Indeterminate nature of the clinical specifiers

The four clinical specifiers of ASD were originally designed to account for the unavoidable heterogeneity of autistic presentation, for example, between nonverbal and hyper-verbal individuals, while preserving the category. These specifiers now exacerbate the heterogeneity of the individuals included in this spectrum, transforming the autism diagnosis into a category as vague as “intellectual disability” and “neurodevelopmental disorders”. A common characteristic of the four clinical specifiers is their dimensional, quantitative, and clinically nonspecific nature. Moreover, there are no constraints on how the qualitative properties of the seven criteria are modified according to the expression of each of the specifiers, which misses a major opportunity to increase specificity. For example, a dissociation between advanced knowledge of letters and numbers and poor pragmatic use of verbs would contribute qualitative information to a quantitative ‘’language” specifier [27].

## Conceptual ambiguity favors heterogeneity

Two conceptual sources of imprecision may further contribute to the current heterogeneity of the autism spectrum: the belief that the clinical threshold for autism is necessarily arbitrary, and the acceptance of any identified neurogenetic or psychiatric condition as a comorbidity, combined with the absence of exclusion criteria or recommended differential diagnoses.

### Is the clinical threshold arbitrary?

A major additional source of heterogeneity in the ASD spectrum is the lowering of the threshold for clinical significance required for the inclusion of individuals who are less different from typical individuals [15]. This escalation in flexibility is frequently justified by the consensus in autism research that clinical thresholds are necessarily arbitrary and/or do not reach reliability among clinicians. The corresponding justification is typically grounded on the philosophical tradition of questioning the status of “natural” boundaries [28]. The description of natural categories separated by objective boundaries has been, since Plato’s illuminating metaphor, compared with “carving nature at its joints”: a butcher does not question the natural boundaries of joints when preparing meat. In defense of the hypothetically arbitrary nature of autism boundaries, this analogy has been ironically transformed by likening the search for a categorical boundary for autism into “carving meatloaf at its joint [29]”. These “joints” were however visible when autism was initially discovered decades ago. However, they disappeared as an effect of the “grinding” of autistic phenotypes into symptoms or traits. The replacement of pattern-like recognition with the use of polythetic criteria in an effort to make such clinical recognition a reliable and objective process has failed. Heterogeneity has introduced itself into the spaces between clinical sub-prototypes and has been authorized by their common inclusion in an overarching, criteria-based category. Hence, the meatloaf “spectrum”.

### Syndromic vs. non-syndromic autism

Although the reported increase in prevalence of autism-like syndrome in a limited number of identifiable neurogenetic syndrome (e.g., Fragile X, Williams syndrome) or identified copy-number variations (e.g., 16p11.2) is recognized, it has also been demonstrated that *any* neurodevelopmental condition accompanied by a certain degree of intellectual disability and behavioral issues increases the probability of satisfying certain autism criteria [30]. The syndromic/non-syndromic distinction has been questioned by some on the basis that today’s non-syndromic autistic presentations will be tomorrow’s syndromic ones, following new discoveries. Waiting for this promised land, the bundling of non-syndromic and syndromic autism assumes external validity for the entire spectrum of discoveries made in patients and animal models with an identified condition sometimes comorbid with autism-like presentations. However, this contention is not supported by the phenotypic dissimilarity between autism with and without penetrant de novo genetic variants [31], nor by the mechanistic differences between the strong effects of reliably identified de novo mutations, on the one hand, and the additive weak effects associated with common variants [13, 32], on the other. In addition, the multiple mutations and pathogenic pathways associated with syndromic autism are only rarely/exceptionnaly [33] traced in non-syndromic autism.

### Are “autistic traits” autistic?

The concept of an autistic trait and the demonstration that “autistic traits are continuously distributed throughout the general population [34]” through instruments such as the Autism Screening Questionnaire [35] and Social Responsiveness Scale [36] has led to the flowering of multiple studies associating autistic traits with nonmedical conditions (e.g., masculinity) [37], separate diagnoses (e.g., anorexia) [38], or in people exposed to a myriad of supposed etiological factors (e.g., cesarean birth) [39]. The increase in the number of such studies during the last decade has been twice as large as for the total number of empirical studies of autism (Fig. 1c,d). Studies reporting autistic traits in a large number of psychiatric or neurological conditions consider them by default as *autistic* traits rather than *socialization features* associated with a particular, non-autistic condition. Are these “autistic traits” themselves autistic? The answer is “no” if they are extracted from the pattern they compose in combination with other traits. All striped animals are not tigers, and all stripes are not *tiger* stripes.

## Disentangling potentially artifactual from genuine heterogeneity

There is, however, heterogeneity that plausibly belongs to the autism signal when the kinship between a prototypical clinical presentation and an altered version is biologically validated. Examples of this include developmental transformations, some (but not all) variations in presentation according to intelligence and language level, and the familial aggregation of autism subtypes.

### Developmental transformation

Removing variation due to age by a de-confounding procedure that integrates the time course in the sign characteristic is likely to remove at least part of the heterogeneity introduced by one of the dominant sources of population variation [40]. However, the developmental transformation of autistic signs [41], while generally trending towards a smaller difference from typicality with age [42], is not a continuous process. Numerous signs in the area of repetitive behaviors and restricted interests present their own developmental course [43], such as, for example, “hand leading”, which is used by a child to nonverbally indicate what he wants. It therefore combines an atypical manner of requesting (positive for the “abnormal social approach” sign) with a specific language-specifier value (speechless plateau), nonverbal intelligence (in the normal range), and a certain age range (2–5 years). Similarly, hand flapping, lateral glances, the absence of overt joint attention, and even most self-injurious behaviors have a “golden age”. These considerations would justify the coupling of age of occurrence with specifier values, and qualitatively defined categorical signs, which could increase the capacity of the clinician to recognize an autistic feature.

### Does familial aggregation of autism subtypes and other psychiatric diagnoses validate autism heterogeneity?

Studies of first-degree relatives of autistic people demonstrate an increased prevalence of cognitive, motor, and psychiatric differences relative to the general population [44]. Familial aggregation of multiple presentations encompassed under the autism spectrum category range from discrepant autism subtypes (e.g., with and without Speech Onset Delay (SOD)  [45]) in siblings to subthreshold atypicalities or a “broader autism phenotype”. At its most extreme, there is a familial co-occurrence of conditions which minimally overlap with the autism phenotype and are clinically considered as differential diagnoses at the phenotypic level, such as specific language impairment [46], or as an unambiguously different type, such as mood disorders [47].

Such familial aggregation validates a certain mechanistic relationship between a prototypical and less prototypical presentation of autism. However, it should not result in encompassing any presentation with a trivial resemblance to autism under a “subthreshold” or “trait” dimension. The independence between genetic alterations and the resulting phenotype associated with them is a well-accepted trivial finding in behavioral genetics. For example, the 22q11.2 deletion syndrome shows variable penetrance and is associated with multiple, phenotypically unrelated psychiatric presentations [48]. In contrast, a minimal variation of the dominant social phenotype in any condition or in the typical population can still be labeled “autistic” [34], even in situations in which the relationship with the full-blown phenotype is unproven—another example of an unfounded “autism exception”.

## Acknowledging the effect of artifactual heterogeneity in clinical settings and research programs

The decrease in effect-sizes in neurocognitive autism research over time is likely due to increased artifactual heterogeneity, which affects our ability to construct neurobiological models of autism. We propose that these problems may be mitigated by modifying the diagnostic criteria and prevalent research strategies.

### Re-building autism subgroups from the recognition of its most prototypical forms

There is more information in the brains of autism experts than that provided by diagnostic instruments. Thus, new criteria should be built from such expertize by decomposing the phenotype of a prototypical population into the qualitative signs that contribute to recognizing autism, which do not coincide with the DSM signs. Experts *recognize* more reliably “frank” autism than any of the diagnostic instruments developed to operationalize this diagnosis using check-list criteria [49]. Importantly, this reliability was independent of age, IQ, and level of functioning. The top-down search for behaviors corresponding to criteria during a diagnostic process is intrinsically more inclusive that the bottom-up recognition of the behavioral patterns that such criteria are based on. Any forthcoming revision of the diagnostic criteria for autism should also restrict the current number of combinatorial possibilities of the clinical specifiers. Instead, it may be beneficial to associate specific combinations of values of these specifiers with specific clinical subtypes.

### Study autism subgroups separately, then compare them

Instead of an *a priori* assumption that all presentations of ASD represent the same condition, it would be beneficial to study potential autism subgroups separately and merge them only if they are similar for targeted variables. Beyond non-syndromic autism with and without SOD, candidate subgroups include syndromic autism, and validated and non-validated subthreshold individuals. Excluding the aspect of speech from the diagnostic criteria accounts for much heterogeneity and increases the risk of losing the information conveyed by speech to the diagnosis. Having or not a history of SOD has a lifelong impact, not only on language and speech [50] but also on the nature of peaks of abilities [51], intelligence subtest profiles [52], motor difficulties [53], domains of interest [54], lateralization of brain structures [55] and functions [56], gyrification [57], white matter [58], and neural activity during speech-like processing [59], which are unavoidably blurred when the two subgroups are analyzed together.

Other possible combinations of IQs, co-occurring conditions, and speech levels or histories may also define relevant subgroups, each with its own neurological and genetic correlates and co-existing symptoms. The absence of such precaution results in the dilution of biological or neurocognitive markers, which are only evident in prototypical individuals, representing the center of a category or subcategory. This dilution biases meta-analyses in favor of type 2 errors [60], closing avenues opened in the first years of autism research [61]. The assumption that co-variation of specifier values in the target analyses will separate their role in the variables under study is only tenable “everything else being equal”, which is wrong if studies inadvertently collapse differently heterogeneous subgroups, known as Simpson’s paradox [62].

### Research autism before the autism spectrum

Conserving a distinct line of research dedicated to prototypical autism is still justified, whereas it is at risk of disappearing under the current spectrum approach to diagnosis. For example, studying the gradual improvement of socio-communicative signs between preschool and school age in children with an initial prototypical presentation provides information on the temporal characteristics of these signs [63]. Such knowledge would help improve the specificity of these signs and their contribution to retrospective diagnoses. It is also necessary to preserve a threshold of qualitative similarity with the prototypical autism phenotype, in addition to the non-specific severity threshold.

### Re-conceptualize autistic signs/traits

The relationship, either mechanistic or phenotypic, between “autistic traits” and autism, implied by the use of the term “autistic” trait rather than “social” or “repetitive” trait, should be scientifically validated, despite some potentially superficial resemblance. Their “autistic” quality should be ascertained by a) their *qualitative* aspects, b) their *co-occurrence* in the prototypical condition, and c) their *contextual validation*, as the demonstration of a previous, developmentally anterior, above-threshold presentation. Trait studies in the general population or certain non-autistic conditions are not informative about above-threshold autism unless the trait-autism link is validated, revealing the weak overlap between genetic proximity and phenotypic similarity [64]. Until there is such validation, “autistic traits” are not yet “autistic”. *The very notion of “autistic traits”, as in “are there autistic traits in the condition X”  may be flawed outside of a context in which their autistic nature is validated*.

## Conclusion

The widely acknowledged heterogeneity of the autism spectrum is not a biological fact of nature. Neither does it have the force of a scientific fact resulting from empirically and logically sound research. Our current notion of autism is partly a result of our ignorance, reinforced by non-specific criteria and the longing for a consensus. The disappearance of the differential diagnosis of autism as a research question, the unfounded but consensual assimilation of autism and autistic traits, and meta-analyses that condense results contaminated by premature assumptions into apparent truth all promote a lasting mechanism to produce null findings. The time has come to usher research on autism towards prototypical individuals and to limit the heterogeneity of autism to a situation in which the variants of the autism phenotype have a traceable link with prototypical autistic individuals.
